# Effects of nitrogen and phosphorus additions on soil microbial biomass and community structure in two reforested tropical forests

**DOI:** 10.1038/srep14378

**Published:** 2015-09-23

**Authors:** Lei Liu, Per Gundersen, Wei Zhang, Tao Zhang, Hao Chen, Jiangming Mo

**Affiliations:** 1 Key Laboratory of Vegetation Restoration and Management of Degraded Ecosystems, South China Botanical Garden, Chinese Academy of Sciences, Guangzhou 510650, China; 2 State Key Laboratory of Urban and Regional Ecology, Research Center for Eco-Environmental Science, Chinese Academy of Sciences, Beijing 100085, China; 3 Department of Geosciences and Natural Resource Management, University of Copenhagen, Rolighedsvej 23, DK-1958 Frederiksberg C, Denmark; 4 Institute of Tropical Pratacultural Science, Zhanjiang Normal University, Zhanjiang, China; 5 University of Chinese Academy of Science, Beijing, China

## Abstract

Elevated nitrogen (N) deposition may aggravate phosphorus (P) deficiency in forests in the warm humid regions of China. To our knowledge, the interactive effects of long-term N deposition and P availability on soil microorganisms in tropical replanted forests remain unclear. We conducted an N and P manipulation experiment with four treatments: control, N addition (15 g N m^−2^·yr^−1^), P addition (15 g P m^−2^·yr^−1^), and N and P addition (15 + 15 g N and P m^−2^·yr^−1^, respectively) in disturbed (planted pine forest with recent harvests of understory vegetation and litter) and rehabilitated (planted with pine, but mixed with broadleaf returning by natural succession) forests in southern China. Nitrogen addition did not significantly affect soil microbial biomass, but significantly decreased the abundance of gram-negative bacteria PLFAs in both forest types. Microbial biomass increased significantly after P addition in the disturbed forest but not in the rehabilitated forest. No interactions between N and P additions on soil microorganisms were observed in either forest type. Our results suggest that microbial growth in replanted forests of southern China may be limited by P rather than by N, and this P limitation may be greater in disturbed forests.

Atmospheric nitrogen (N) deposition has been increasing globally, especially in the warm and humid climatic zone in Asia^1^. In some tropical and subtropical forests of southern China, inorganic N deposition is 30–73 kg N ha^−1·^yr^−1^ and among the highest in the world^2^. Adverse effects of enhanced N deposition, including soil acidification, nutrient imbalance, loss of plant diversity, and even forest decline have been recorded from temperate forests of North America and Europe^3^,^4^,^5^,^6^. In contrast to the temperate forests, which are often N-limited under natural conditions, tropical forests are mainly P-limited, with old weathered soils that are often highly acidic and low in base cation availability^7^,^8^. Fertilization experiments demonstrated that adding N can increase P cycling. Nevertheless, the increase in P cycling induced by N input is insufficient, and the affected tropical forests may become P limited^9^. Studies of old growth forest or forests with minor disturbances in southern China indicate N saturation (e.g. elevated N leaching) resulting from the increased N deposition^10^, and P limitation of soil microorganisms^11^. A 30-yr time series of plant chemistry and production in these forests also revealed signs of progressive P limitation, including decreases in available soil P, increases in N/P ratios in leaves and litterfall, increases in litterfall amount, and decreases in aboveground primary production^12^.

Soil microorganisms play key roles in ecosystems and mediate many ecological processes that are crucial to ecosystem functioning, including decomposition processes^13^ and nutrient cycling^14^. The biomass and activity of microorganisms is typically thought to be constrained by the availability and quality of carbon (C)^15^. However, soil nutrient availability can also influence soil microbial growth and activity^16^. A theoretical model revealed that while total C flow may be limited by the functioning of the exoenzyme system, actual microbial growth may be N limited^17^. Gallardo and Schlesinger (1994) also suggested that microbial P limitation may be common in highly weathered soils in which P tends to be bound to iron or aluminum sesquioxides^18^. Some studies have shown structurally distinct microbial communities, as indexed by phospholipid fatty acids (PLFAs), under high N deposition or P addition^11^,^19^. Gusewell & Gessner (2009) reported a higher relative abundance of fungi on cellulose when P was limiting, whereas bacteria were more abundant when N was limiting in a microcosm experiment^20^. However, effects of nutrient addition on soil microbial communities are inconsistent in the literature^21^, and little is known regarding if and how interactions between N and P influence soil microbial communities in tropical forests.

To fill in this gap in our knowledge, we established a full factorial N and P addition experiment in three forest types at the Dinghushan Biosphere Reserve (DHSBR) in southern China in 2007. The three forest types included an old-growth tropical forest, a disturbed (planted pine forest with recent harvests of understory vegetation and litter), and a rehabilitated forest (planted with pine but mixed with broadleaf returning by natural succession). We have previously reported results for the old-growth forest^21^. As expected, additions of P increased soil microbial biomass and additions of N reduced soil microbial biomass in the old-growth forest; however, these effects were transient and disappeared over longer periods^21^. In this paper, we report the results from the other two forest types.

Unlike the old-growth forest, which is an undisturbed forest with minimal direct human impacts, and has been protected by monks for more than 400 years, the disturbed forests (the mixed and pine forests) originated from clear-cuts in the 1930 s and subsequent pine plantation establishment. These kinds of disturbed forests cover more than half of the total forest area in subtropical and tropical China^22^,^23^. The rapid economic development in Guangdong south-eastern China has led to major land-use changes and various levels of deforestation^24^ that have caused serious environmental problems, such as severe soil erosion, loss of wildlife habitat, and loss of forest cover. In order to reduce these environmental problems and prevent further degradation, large areas have been reforested with a native pine species (*Pinus massoniana* Lamb). Cutting trees is usually prohibited in the ‘new’ forests, but harvesting of understory and litter is often allowed to satisfy local fuel needs^25^. To the best of our knowledge, the effects of these major nutrient removals from human disturbance on ecosystem processes are poorly studied^22^,^25^ and particular information regarding the long-term effects on soil microorganisms is lacking.

We found no short-term (30 months) effect of P addition on soil microbial biomass in these two forest sites^11^, but based on litter decomposition studies we observed signs that microorganisms in human disturbed forests in tropical China are N limited^25^,^26^. Previous studies also showed that there was a lag effect of P addition on soil microbial biomass in old-growth forests in the same area (effect on soil microbial biomass significantly at 30-month, but not significantly at 18-month following P addition)^27^. Therefore, the objectives of this study were to: 1) determine whether there was an interactive effect of N and P addition on soil microorganisms, 2) explore the long-term effects of N and P addition on soil microorganisms, and 3) determine whether the effects of N and P addition on the soil microbial community were mediated by land-use history. We hypothesised that N is a limiting factor for microbial growth in these reforested forests (disturbed and rehabilitated), and that P may also limit microbial growth in the disturbed forest because of the continuous removal of P by harvesting.

## Results

### Soil properties

There was no significant difference in the chemical parameters for soil between the disturbed and rehabilitated forests, except for SMC and soil NO_3_^–^–N concentrations (Table 1). Nitrogen addition did not change soil properties, except for NO_3_^–^–N and NH_4_^+^-N concentrations. Soil NO_3_^–^–N concentrations were also altered in the P-addition plots. Soil available P was significantly elevated in the P- amended plots in both the disturbed and rehabilitated forests (Table 1). Soil net mineralization rate and nitrification rate were different between forest types, and were lower in the disturbed than the rehabilitated forest. Statistical analysis from a three-way analysis variance (ANOVA) showed that there were no significant interactions between N and P additions on the chemical parameters of soil (Table 1).

### Soil microbial biomass

**Figure 1:**
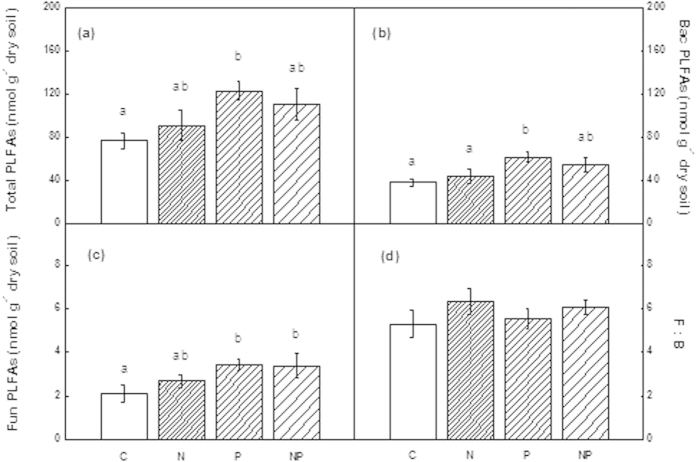
Comparisons of soil microbial PLFAs between treatments in the disturbed forest. Data from June 2011. C: control, N: nitrogen addition, P: phosphorus addition, NP: nitrogen and phosphorus addition. F:B indicates the ratio of fungal to bacterial PLFAs presented as percentage. Significant differences (*p* < 0.05) among treatments are indicated by different letters. Error bars represent SE (n = 5).

**Figure 2:**
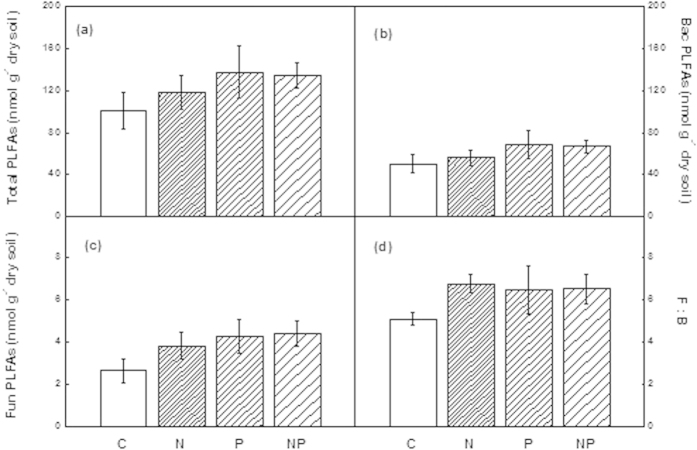
Comparisons of soil microbial PLFAs between treatments in the rehabilitated forest. Data from June 2011. C: control, N: nitrogen addition, P: phosphorus addition, NP: nitrogen and phosphorus addition. F:B indicates the ratio of fungal to bacterial PLFAs presented as percentage. Significant differences (*p* < 0.05) among treatments are indicated by different letters. Error bars represent SE (n = 5).

There was no significant effect on soil microbial biomass after N addition in either of the forest types. Response of soil microbial biomass to P addition, however, varied depending on forest type (Figs 1 and 2). P addition significantly increased microbial biomass, including total PLFAs, bacterial PLFAs, and fungal PLFAs in the disturbed forest, but not in the rehabilitated forest. Soil microbial biomass did not respond to NP addition in either forest, except for fungal PLFAs, which was significantly increased after NP addition in the disturbed forest (Fig. 1). Although there was no significant treatment responses in the rehabilitated forest (Fig. 2), the trend in the responses were identical to that of the disturbed forest (Fig. 1).

### Soil microbial community

**Figure 3:**
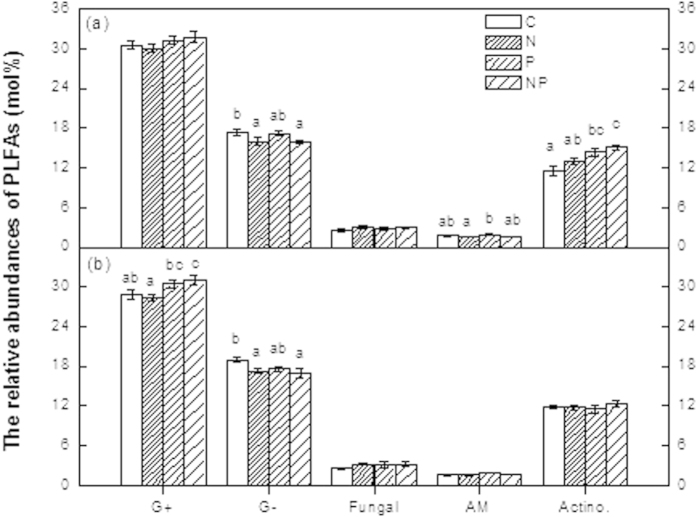
Relative abundances of the individual PLFAs (mol%) in soils from the (a) disturbed and (b) rehabilitated forest. G^+^: the proportion of gram-positive bacterial PLFAs; G^–^: the proportion of gram-negative bacterial PLFAs; Fungi: the proportion of fungal PLFAs; AM: the proportion of AM fungal PLFAs; Actino: the proportion of actinomycetes PLFAs. Significant differences (*p* < 0.05) among treatments are indicated by different letters. Error bars represent SE (n = 5).

**Figure 4:**
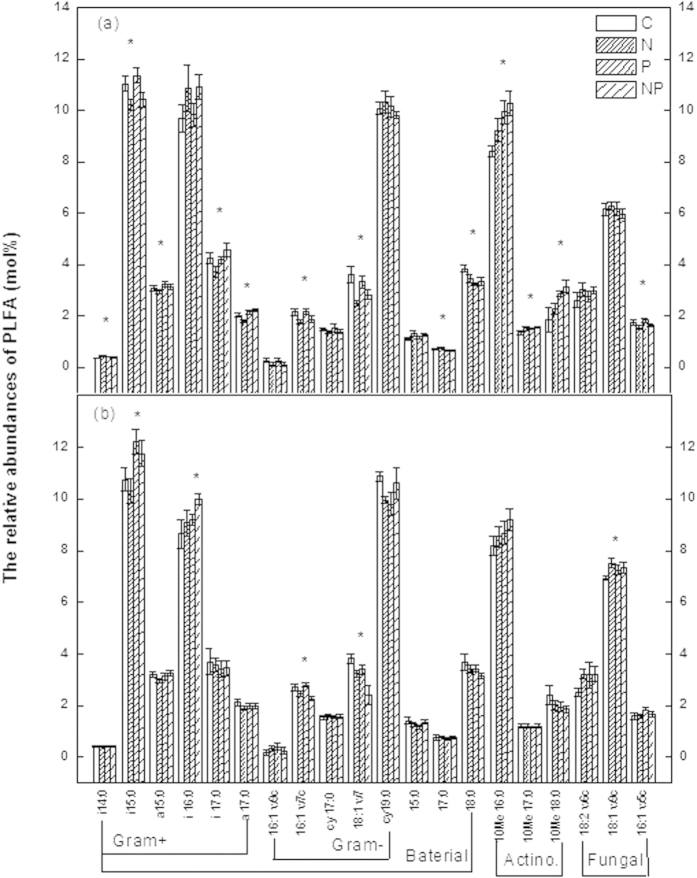
The relative abundances of the individual PLFAs (mol%) in soils from the (a) disturbed and (b) rehabilitated forest. C: control, N: nitrogen addition, P: phosphorus addition, NP: nitrogen and phosphorus addition. Significant differences (*p* < 0.05) among treatments are indicated by *. Error bars represent SE (n = 5).

The mean abundance of gram-negative bacteria PLFAs were significantly decreased in the N- amended plots in both forests (Fig. 3). The lower abundance of gram-negative bacteria PLFAs in the N- amended plots was mainly reflected in the relative abundance of the individual PLFAs 16:1w7c and 18:1w7c (Fig. 4). Moreover, the ratio of cyclopropyl fatty acid to their precursors (cy17:0/16:1w7c and cy19:0/18:1w7c) was also significantly affected by N addition in both forests (Table S1, S2). Conversely, the mean abundance of actinomycetes PLFAs in the P- amended plots was significantly increased in the disturbed forest, but not in the rehabilitated forest (Fig. 3). There were no interactions between N and P addition on soil microbial community structure, except for a few individual PLFAs (Appendix S1, S2).

In addition, the mean abundance of gram-positive PLFAs was significantly higher in the P- amended plots than in the N- amended plots in the rehabilitated forest, whereas the mean abundances of arbuscular mycorrhizal (AM) PLFAs was significantly higher in the P-addition plots compared with the N-addition plots in the disturbed forest (Fig. 3).

**Figure 5:**
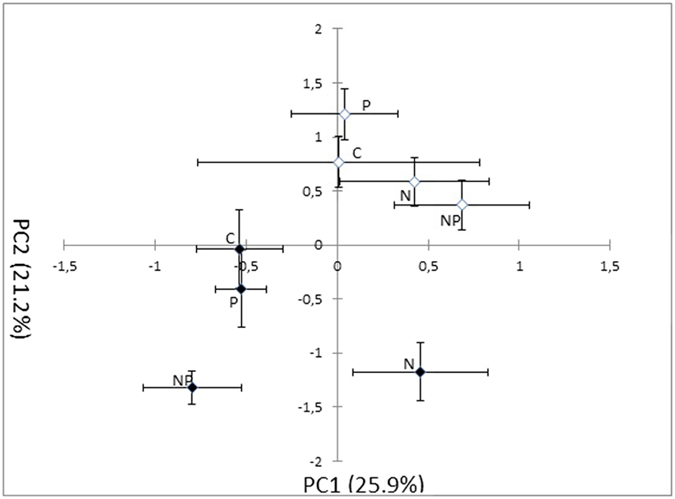
Principal component analysis (PCA) of PLFA pattern in the disturbed and rehabilitated forests. *Filled* disturbed forest, *open* rehabilitated forest.

The first two axes produced by principal components analysis (PCA) accounted for 47.1% of the total variation in the PLFA profile; the forest types were separated along the PC1 and PC2 axes (Table 2, Fig. 5) indicating differences in microbial community structure. Nitrogen addition affected community structure significantly by shifting along the PC2 axis of the N-addition plots compared to the control plots (Fig. 5, Table 2). When comparing the effect of soil chemical properties on soil microbial community composition, soil pH and inorganic N had no effect on soil microbial community composition. Soil organic carbon (SOC) and available P significantly influenced soil community composition (Table 3).

## Discussion

### Interactive effects of N and P addition

As interactive effects of combined N and P enrichment have been frequently observed in many terrestrial ecosystems^1^, we expected that simultaneous N and P addition would produce notable interaction effects on soil microbial biomass or community composition in these tropical forests. Conversely, the expected interaction effects were not found in the present study (Appendix S1, S2). Similarly, interaction effects were not encountered in an old-growth forest in the same area^21^. Fanin *et al.* (2015) also reported that no evidence for N and P interactions on soil microbial structure and functioning in an Amazonian rain forest^28^. Compared to other resources, however, N is relatively abundant at our study forests due to high inorganic N deposition^2^. As a result, N may not be a limiting nutrient for soil microorganisms, and consequently, additional N fertilization may not increase soil microbial biomass or activities.

### Effect of N addition

Nitrogen addition generally had a negative effect on microbial biomass in both field and lab-based studies^29^. A number of mechanisms have been proposed for the decline in microbial biomass under N fertilization, such as direct inhibition, indirect effect due to magnesium or calcium deficiency caused by soil acidification, or alteration of C availability^29^. On the other hand, aboveground litter production usually increases and litter quality may improve under N fertilization^30^,^31^. In this case, soil microbial biomass may increase because of alleviated C and N limitation as shown by results from temperate forests, which are typically N-limited under natural conditions^32^,^33^. Contrary to our original hypotheses, no significant effect of N addition on soil microbial biomass was found over a long-term (52-month) period in either forest (Figs 1 and 2). Li *et al.* (2015) also reported that N addition did not significantly affect microbial biomass in a secondary tropical forest of China^34^. The lack of a clear effect of N addition on soil microbial biomass, as mentioned above, is unlikely because of the relatively high N status in the studied forests.

Although long-term N addition had no effect on microbial biomass, it had an impact on microbial community composition. Similar results were reported in the study of three Hawaiian forests^35^. We found that N addition altered microbial community structure by significantly decreasing the relative abundance of gram-negative bacteria (e.g. 16:1w7c and 18:1w7c) in both forests. Gilliam *et al.* (2011) found that the gram-negative PLFA 18:1n7c (gram-negative bacteria) was predominant in soils with the highest rates of net nitrification at Fernow Experimental Forest, a central Appalachian hardwood forest in West Virginia, USA^16^. In the present study, N addition actually decreased the relative abundance of gram-negative bacteria with lower net nitrification, which may include NH_4_-oxidizing and NO_2_-oxidizing (ammonia-oxidizing and nitrite-oxidizing) gram-negative bacteria. In a parallel investigation in an old growth forest, a similar negative response of gram-negative bacteria to N addition was observed^21^. These results indicated that N is unlikely a limiting factor, but rather a negative factor for soil microorganisms in these forests.

We further observed a shift in bacterial communities in response to N fertilization as indicated by an increase of the cy:pre ratio (cy 17:0/16:1w7c and cy19:0/18:1w7c), which is an indicator of physiological stress or C limitation^36^,^37^. Higher values of these ratios have been associated with decreased bacterial growth rate and increased C limitation^37^,^38^. In this study, the cy:pre ratios were higher in N-amended plots than in control plots for both forests (Appendix S1, S2). These results indicated that the microbial community in N-amended plots may be in physiological stress, and be C limited in these reforested tropical forests. Fanin *et al.* (2015) also reported that the responses of microbial community structure to N fertilization were controlled by C availability in an Amazonian rain forest^28^.

### Effect of P addition

The observed strong microbial responses to P addition are in line with the results of previous experiments in other tropical lowland forest in France Guiana, Costa Rica, and Panama^28^,^39^,^40^. There are several mechanisms that cause P limitation in terrestrial ecosystems. In the present study, the N deposition reaches 30–70 kg N ha^−1^·yr^−1^, such high N deposition in this region may increase N availability, but also aggravates P deficiency in these old weathered soils^2^. Therefore, the relieved P constraints could increase soil microbial biomass, and a previous study found that microbial utilization of soil C in these tropical forests was P limited^39^. However, the P limitation of microbial biomass was only found in the disturbed forest and differed from the short-term results, which did not indicate a significant effect of P addition on microbial biomass in either forest 30-month after P addition^11^.

The lag effect of P addition on soil microbial biomass in this study is possibly that soil microorganisms are C limited in the initial years following P addition. The annual total litterfalls increased, and thus alleviated C limitation for soil microorganisms under P fertilization^11^. The lag effect of P addition on soil microbial biomass was also found in a nearby old growth forest (no effect after 18-month, but significant effect after 30-month)^11^,^27^. Fanin *et al.* (2015) recently showed that heterotrophic microbial communities are simultaneously limited by P and available C^28^. Ehlers *et al.* (2010) also observed P limitation of microbial growth after a combined C and N addition in a native soil from western Kenya^41^.

The different response of soil microbial biomass to P addition between these two forests may be related to the differences in tree species composition, and soil nutrient status caused by different land-use practices. In the present study, *Pinus massoniana* was the dominant tree in the disturbed forest (>80% of basal area) in comparison to the rehabilitated forest (<40% of basal area)^11^. The concentration of N and P in the pine needles was higher in the rehabilitated forest than in the disturbed forest^26^. Moreover, N addition increased mass loss rate and C release rate, but suppressed the release rate of N and P from decomposing needles in both disturbed and rehabilitated forests^26^. Therefore, there was less N and P released from litter decomposition in the disturbed forest than in the rehabilitated forest. These results indicated that soil microbial growth maybe more P-limited in disturbed forests because of the low P release rate from litter decomposition. In addition, the understory and litter harvesting practice removed 44 to 73% of the nutrients (N, P, K, Ca, and Mg) from the annual production of litter and understory biomass until the late 1990 s^22^. Therefore, P was one of the limiting factors in the disturbed forest rather than in the rehabilitated forest.

## Conclusions

In conclusion, we found that there were no interaction effects of N and P addition on soil microbial biomass or community composition. N availability was not a limiting factor for microbial growth in the reforested sites of the studied region and the disturbed forest may be more sensitive to nitrogen addition. However, P availability was one of the limiting factors for microbial growth in the disturbed forest, but not in the rehabilitated forest. These results imply that nutrient limitation on soil microorganisms depend on the changes in tree species composition and soil nutrient status caused by the degree of human disturbance and land-use practices under high N deposition. In addition, our results suggested that there is a lag time in the effect of P addition on soil microorganisms in these tropical forests.

## Materials and Methods

### Site description

This study was conducted in Dinghushan Biosphere Reserve, which covers an area of 1,200 ha and is located in the central part of Guangdong Province, south China (112°10′ E, 23°10′ N). This Reserve is about 90 km west of metropolitan Guangzhou (10 million inhabitants). In the Reserve, we have identified two types of reforest forests: a mixed pine and broadleaf forest (rehabilitated) and a pine forest (disturbed). The rehabilitated forest, at about 200 m above sea level (asl) occupied approximately 50% of the Reserve, and the disturbed forest, at about 50–200 m asl occupied approximately 20% of the reverse^25^. The remaining 30% of the Reserve is covered by the undisturbed old growth forest where a parallel study was completed by Liu *et al.* (2013)^21^. Both rehabilitated and disturbed forests originate from the clear-cuts of the 1930 s and subsequent pine plantation establishment. They were under continuous human disturbance (generally the harvesting of understory and litter) from 1930 to 1956 (rehabilitated forest) and 1998 (disturbed forest). In the rehabilitated forest, after cessation of the disturbance, colonization from the natural dispersal of regional broadleaf species altered its plant community. Thus, these forests vary both in the level of human impacts, as well as stages of succession, site conditions, and species assemblages^25^. Dominant tree species in the rehabilitated forest are *Pinus massoniana*, *Schima superba* Chardn. & Champ., *Castanopsis chinensis* Hance, *Craibiodendron kwangtungense* S. Y. Hu, *Lindera metcalfiana* Allen, and *Cryptocarya concinna* Hance, whereas, *P. massoniana* remained the dominant tree in the disturbed forest^11^ Further details on the tree structure at the sites can be found in Liu *et al.* (2012). The selected sites of the two forest types were approximately 4 km from each other.

The Reserve has a monsoon season and humid climate. The average annual precipitation of 1,927 mm has a distinct seasonal pattern with 75% from March through August and only 6% from December through February^25^. The mean annual temperature is 21 °C, with the coldest and warmest months being January (12.6 °C) and July (28.0 °C), respectively. Annual mean relative moisture is 80%. The N deposition measured as inorganic N in throughfall was 24 and 26 kg N ha^−1^·yr^−1^ in 2004 and 2005 for the rehabilitated and disturbed forests, respectively, with an additional input of 15–20 kg N ha^−1^·yr^−1^ as dissolved organic N^42^. The soil in the Reserve is lateritic red earth formed from sandstone^25^. The soil depth ranges from 30 to 60 cm (to the top of the C horizon) in the rehabilitated forest and is generally less than 30 cm in the disturbed forest^25^.

### Experimental treatment

In 2007, four treatments (each in five replicates) were established in both forests: Control, N-addition (15 g N m^−2^·yr^−1^), P-addition (15 g P m^−2^·yr^−1^), and NP-addition (15 g N m^−2^·yr^−1^ plus 15 g P m^−2^·yr^−1^). Each of the 20 plots was 5 m × 5 m and surrounded by a 5-m wide buffer strip separating the next plot. Field plots and treatments were laid out randomly. Plots size and fertilizer level were similar to those in the experiment in Costa Rica by Cleveland and Townsend (2006)^43^. Applications of N and P were made as NH_4_NO_3_ and NaH_2_PO_4_ solutions sprayed in two monthly portions below the canopy with a backpack sprayer starting from January 2007 and continuing through the end of this project (June 2011). Fertilizer r was weighed and mixed with 5 L of water for each plot. Each control plot received 5 L of water without fertilizer.

### Field sampling and measurements

Soil sampling of the upper 10 cm was conducted in the warm and wet season, June 2011. From each plot, 5 soil core samples (2.5 cm inner diameter) were collected randomly and combined into one composite sample. The litter layer was carefully removed before sampling. After removing stones and coarse roots, soils were sieved through a 2-mm mesh and divided into two parts, one retained for measuring soil chemical parameters and the other for analysis of microbial biomass and community structure. At the same time, an *in situ* soil-core technique^44^ was used to estimate soil net nitrification rates.

Soil moisture content (SMC) was measured gravimetrically using 10 g of moist soil that was oven dried at 105 °C for 24 h. Soil pH was measured in a 1:2.5 soil/water suspension. Soil organic C (SOC) was determined by dichromate oxidation and titration with ferrous ammonium sulfate. Dissolved organic carbon (DOC) in filtered 0.5 M K_2_SO_4_-extracts of fresh soil was measured with a TOC analyser (TOC-VCPH Shimadzu Corp., Japan). NH_4_^+^-N and NO_3_^–^–N in filtered 2 M KCl-extracts of fresh soil were measured with a flow injection autoanalyser (FIA, Lachat Instruments, USA). Available P concentration was analysed colorimetrically after acidified ammonium persulfate digestion^45^.

Soil microbial biomass and community structure was characterised using phospholipid fatty acid (PLFA) analysis as described by Bossio and Scow (1998)^36^. The abundance of individual fatty acids was determined as nmol per g of dry soil and standard nomenclature was used^46^ (Tunlid *et al.* 1989). Concentrations of each PLFA were calculated based on the 19:0 internal standard concentrations. Frostegård and Bååth (1996) chose a set of fatty acids to represent bacterial PLFAs, out of which i14:0, 15:0, i15:0, a15:0, i16:0, 16:1ω7c, 17:0, a17:0, i17:0, cy17:0, 18:1ω7, and cy19:0 were present in our samples^47^. We calculated the sum of i14:0, i15:0, a15:0, i16:0, a17:0, and i17:0 as an indicator of gram-positive bacteria. In our study, gram-negative bacteria were identified by the PLFAs: 16:1ω7c, cy17:0, 18:1ω7, and cy19:0^48^. The fungi were identified by the PLFA 18:2ω6,9c^47^, and PLFAs 16:1ω5c was used as a marker for arbuscular mycorrhizal (AM) fungi^49^. The actinomycetes were identified by the PLFAs 10Me 16:0, 10Me 17:0, and 10Me 18:0^50^. Other PLFAs, such as 14:0, 16:0, 16:1 2OH, 16:1ω9c, 17:1ω8c, 18:1ω9c, 10Me 19:0, 18:3ω6c, and 20:1ω9c were also used to analyze the composition of the microbial community. The ratio of 18:2ω6,9c to total bacterial PLFAs was used to estimate the ratio of fungal to bacterial biomass (F:B) in soils^47^,^51^.

### Statistical analysis

We used a two-way ANOVA to examine the difference in soil microbial biomass, and F:B ratios among treatments for each forest. These statistical analyses were carried out with SAS for windows version 8 (Analysis of Variance, PROC GLM from SAS). Twenty-six PLFAs were detected, identified, and included in the principal component analysis (PCA) after standardisation for equal unit variance. The test of statistical significance for PCA was run using CANOCO software for Windows 5.0 (Ithaca, NY, USA). The relationships between soil microbial community and soil chemical variables were assessed using Mantel tests with the ecodist package in R. Statistically significant differences were identified as *P* < 0.05, unless otherwise stated.

## Additional Information

**How to cite this article**: Liu, L. *et al.* Effects of nitrogen and phosphorus additions on soil microbial biomass and community structure in two reforested tropical forests. *Sci. Rep.* **5**, 14378; doi: 10.1038/srep14378 (2015).

## Supplementary Material

Supplementary Information

## Figures and Tables

**Table 1:** Effects of forest, N addition, P addition, and two-way interactions of N and P addition on soil properties (0–10 cm depth), n = 5.

**Factors**	**Forest type**		**N addition**		**P addition**		**N** **×** **P**	
	***F***	***P***	***F***	***P***	***F***	***P***	***F***	***P***
SMC	6.08	**<0.05**	2.95	0.09	1.28	0.26	2.76	0.11
NO_3_^–^–N	15.8	**<0.001**	20.3	**<0.001**	10.5	**0.002**	0.04	0.85
NH_4_^+^-N	3.36	0.08	10.5	**0.003**	0.24	0.63	0.59	0.45
pH(H_2_O)	3.88	0.06	3.18	0.08	3.37	0.07	2.55	0.12
SOC	0.31	0.58	1.66	0.21	0.26	0.62	0.15	0.70
Avail P	0.01	0.92	0.28	0.60	92.6	**<0.001**	0.02	0.90
NMR	36.1	**<0.001**	4.01	0.05	6.43	**<0.05**	1.69	0.20
NNR	4.86	**<0.05**	0.77	0.38	0.57	0.45	0.85	0.36

N × P: interactions between N addition and P addition; SMC: soil moisture content; SOC: soil organic carbon; Avail P: available P; NMR: net mineralization rate; NNR: net nitrification rate.

Significant *P*-values (*P* < 0.05) shown in bold face type.

**Table 2:** Effects of forest, N addition, P addition, and two-way interactions of N and P addition on soil microbial PLFAs (n = 5).

**Factors**	**Forest type**		**N addition**		**P addition**		**N** **×** **P**	
	***F***	***P***	***F***	***P***	***F***	***P***	***F***	***P***
Total PLFAs	**4.16**	**<0.05**	0.14	0.71	**7.69**	**0.009**	1.15	0.29
Bacterial PLFAs	4.00	0.05	0.02	0.90	**8.97**	**0.005**	0.91	0.35
Fungal PLFAs	**5.37**	**<0.05**	1.47	0.23	**7.37**	**<0.05**	1.14	0.29
F: B	0.74	0.39	3.35	0.08	0.38	0.54	1.53	0.23
PC1	5.05	**<0.05**	2.44	0.13	0.69	0.41	0.82	0.37
PC2	58.4	**<0.001**	15.9	**<0.001**	0.13	0.72	0.31	0.58

N × P: interactions between N addition and P addition; F:B indicates the percent of ratio of fungal to bacterial PLFAs; PC1 and PC2 indicate the first two axes produced by principal components analysis based on the total variation in the PLFA profile.

**Table 3:** Relationships of soil microbial community composition with soil chemical variables, as revealed by Mantel and partial Mantel tests.

**Explanatory factor**	**Mantel test**		**Partial Mantel test**	
	*r*_*M*_	*P*	*r*_*M*_	*P*
pH	0.073	0.176	—	—
Moisture	−0.093	0.909	—	—
NO_3_^–^ -N	0.090	0.133	—	—
NH_4_^+^-N	−0.096	0.904	—	—
SOC	**0.187**	**0.029**	**0.190**	**0.027**
Available P	**0.110**	**0.016**	**0.115**	**0.01**

Values in bold indicate significant correlations (*p* < 0.05). SOC: soil organic carbon.
