# Supplementary material for: Effects of nitrogen and phosphorus additions on soil microbial biomass and community structure in two reforested tropical forests
Source: Sci Rep. 2015 Sep 23;5:14378. doi: 10.1038/srep14378 (PMC4585765; doi:10.1038/srep14378)
Supplement: Supplementary Information [file srep14378-s1.doc]

**Supplementary information**

**Title:** Effects of nitrogen and phosphorus additions on soil microbial biomass and community structure in two reforested tropical forests

Lei Liu^1, 2^, Per Gundersen^3^, Wei Zhang^1^, Tao Zhang^4^, Hao Chen^1, 5^, Jiangming Mo^1*^

**^1^**Key Laboratory of Vegetation Restoration and Management of Degraded Ecosystems, South China Botanical Garden, Chinese Academy of Sciences, Guangzhou 510650, China.

^2^State Key Laboratory of Urban and Regional Ecology, Research Center for Eco-Environmental Science, Chinese Academy of Sciences, Beijing 100085, China.

^3^Department of Geosciences and Natural Resource Management, University of Copenhagen, Rolighedsvej 23, DK-1958 Frederiksberg C, Denmark.

^4^Institute of Tropical Pratacultural Science, Zhanjiang Normal University, Zhanjiang, China

^5^University of Chinese Academy of Science, Beijing, China

* Corresponding author: Jiangming Mo

Tel.: +86 758 2621187

Fax: +86 758 2623242

Email: [mojm@scib.ac.cn](mailto:mojm@scib.ac.cn)

**Number of tables**: 3

**Number of figures**: 5

**Number of reference**: 51

**Supplementary information:**

**Appendix S1.** Effects of N addition, P addition and two-way interactions of N addition and P addition on soil chemistry parameters and microbial characteristics in disturbed forest, n = 5.

| Factors | N additions | | P additions | | N*P additions | |
| --- | --- | --- | --- | --- | --- | --- |
|  | *F* | *P* | *F* | *P* | *F* | *P* |
| NO_3_^-^-N | **17.0** | **0.0008** | **8.76** | **0.009** | 0.70 | 0.42 |
| NH_4_^+^-N | **11.9** | **0.003** | 0.84 | 0.37 | 0.65 | 0.43 |
| pH | 1.01 | 0.33 | 0.94 | 0.35 | 0.04 | 0.85 |
| SOC | 1.72 | 0.21 | 1.68 | 0.21 | 0.82 | 0.38 |
| Avail P | 0.04 | 0.85 | **50.4** | **< 0.0001** | 0.44 | 0.51 |
| G+ (mol %) | 0 | 0.97 | 3.33 | 0.09 | 0.56 | 0.47 |
| G- (mol %) | **9.36** | **0.008** | 0.06 | 0.81 | 0.03 | 0.87 |
| Fungi (mol %) | 1.82 | 0.20 | 0.05 | 0.82 | 0.24 | 0.63 |
| AM (mol% ) | **5.61** | **0.03** | 1.64 | 0.22 | 0.05 | 0.82 |
| Actino. (mol% ) | 3.84 | 0.07 | **18.9** | **0.0005** | 0.44 | 0.52 |
| i14:0 | 1.76 | 0.20 | 0.10 | 0.76 | 3.31 | 0.09 |
| i15:0 | **10.5** | **0.005** | 0.91 | 0.35 | 0.06 | 0.80 |
| a15:0 | 1.74 | 0.21 | 3.24 | 0.09 | 0.07 | 0.79 |
| i 16:0 | 3.25 | 0.09 | 0.02 | 0.88 | 0.01 | 0.93 |
| i 17:0 | 0.10 | 0.75 | 3.57 | 0.08 | **5.25** | **0.04** |
| a 17:0 | 1.53 | 0.23 | **21.03** | **0.0003** | **7.12** | **0.02** |
| 16:1 w9c | 4.72 | 0.05 | 0 | 0.95 | 0.02 | 0.89 |
| 16:1 w7c | **8.68** | **0.01** | 0.29 | 0.60 | 0.13 | 0.72 |
| cy 17:0 | 1.24 | 0.28 | 0.08 | 0.79 | 0 | 0.97 |
| 18:1 w7 | **12.7** | **0.003** | 0.01 | 0.93 | 1.81 | 0.20 |
| cy19:0 | 0.03 | 0.88 | 0.47 | 0.50 | 0.75 | 0.40 |
| 15:0 | 4.49 | 0.05 | 0.01 | 0.92 | 0.64 | 0.44 |
| 17:0 | 0.27 | 0.61 | **4.78** | **0.04** | 1.74 | 0.21 |
| 18:0 | 1.17 | 0.30 | **7.40** | **0.02** | 4.12 | 0.06 |
| 10Me 16:0 | 2.10 | 0.17 | **10.17** | **0.006** | 0.34 | 0.57 |
| 10Me 17:0 | 4.08 | 0.06 | 4.03 | 0.06 | 0.65 | 0.43 |
| 10Me 18:0 | 1.60 | 0.22 | **11.1** | **0.004** | 0.08 | 0.78 |
| 18:1 w9c | 0.02 | 0.90 | 0.44 | 0.52 | 0.48 | 0.50 |
| PC1 | **4.67** | **0.04** | 3.94 | 0.06 | 2.39 | 0.14 |
| PC2 | **5.19** | **0.04** | **8.35** | **0.01** | 0.04 | 0.84 |

**Note:** N * P: interactions between N addition and P addition; SOC: soil organic carbon; Avail P: available P. F: B indicates the ratio of fungal to bacterial PLFAs. G^+^: the proportion of gram-positive bacterial PLFAs; G^-^: the proportion of gram-negative bacterial PLFAs; Fungi: the proportion of fungal PLFAs; AM: the proportion of AM fungal PLFAs; Actino.: the proportion of actinomycetes PLFAs. Results are from two-way factorial ANOVA for the soil microorganism variables.

**Appendix S2.** Effects of N addition, P addition and two-way interactions of N addition and P addition on soil chemistry parameters and microbial characteristics in rehabilitated forest, n = 5.

| Factors | N additions | | P additions | | N*P additions | |
| --- | --- | --- | --- | --- | --- | --- |
|  | *F* | *P* | *F* | *P* | *F* | *P* |
| NO_3_^-^-N | **5.88** | **0.03** | 3.08 | 0.10 | 1.83 | 0.19 |
| NH_4_^+^-N | 2.16 | 0.16 | 1.74 | 0.21 | 0.13 | 0.73 |
| pH | 2.15 | 0.16 | 2.48 | 0.13 | 3.88 | 0.07 |
| SOC | 0.18 | 0.67 | 0.83 | 0.38 | 0.34 | 0.57 |
| Avail P | 0.73 | 0.41 | **40.0** | **< 0.0001** | 0.55 | 0.47 |
| cy19:0/18:1 w7 | **7.24** | **0.01** | **5.07** | **0.04** | 4.32 | 0.05 |
| G+ (mol %) | 0 | 0.99 | **10.7** | **0.005** | 0.63 | 0.44 |
| G- (mol %) | **6.10** | **0.03** | 3.31 | 0.09 | 1.15 | 0.30 |
| Fungi (mol %) | 1.30 | 0.27 | 1.12 | 0.31 | 1.02 | 0.33 |
| AM (mol% ) | 0.96 | 0.34 | 2.40 | 0.14 | 1.10 | 0.31 |
| Actino. (mol% ) | 0.72 | 0.41 | 0.04 | 0.84 | 0.73 | 0.41 |
| i14:0 | 0.11 | 0.74 | 0.24 | 0.63 | 0 | 0.99 |
| i15:0 | 0.85 | 0.37 | **9.05** | **0.008** | 0 | 0.99 |
| a15:0 | 0.26 | 0.62 | 0.89 | 0.36 | 2.56 | 0.13 |
| i 16:0 | 2.18 | 0.16 | 3.31 | 0.09 | 0.19 | 0.67 |
| i 17:0 | 0.01 | 0.92 | 0.38 | 0.55 | 0.05 | 0.83 |
| a 17:0 | 1.10 | 0.31 | 0.12 | 0.73 | 1.14 | 0.30 |
| 16:1 w9c | 0 | 0.96 | 0.46 | 0.51 | 2.11 | 0.17 |
| 16:1 w7c | **14.8** | **0.001** | 0.11 | 0.75 | 1.70 | 0.21 |
| cy 17:0 | 0.35 | 0.56 | 0.01 | 0.94 | 0.16 | 0.69 |
| 18:1 w7 | **10.9** | **0.005** | **6.85** | **0.02** | 0.66 | 0.43 |
| cy19:0 | 0.02 | 0.89 | 0.31 | 0.59 | **4.97** | **0.04** |
| 15:0 | 0.06 | 0.82 | 0.73 | 0.41 | 3.33 | 0.09 |
| 17:0 | 0.28 | 0.61 | 0.60 | 0.45 | 0.05 | 0.83 |
| 18:0 | 2.10 | 0.17 | 1.90 | 0.19 | 0 | 0.95 |
| 10Me 16:0 | 1.09 | 0.31 | 1.99 | 0.18 | 0.04 | 0.85 |
| 10Me 17:0 | 0.17 | 0.69 | 0.07 | 0.80 | 0.03 | 0.86 |
| 10Me 18:0 | 0.92 | 0.35 | 1.61 | 0.22 | 0.34 | 0.57 |
| 18:1 w9c | 3.29 | 0.09 | 0.19 | 0.67 | 1.73 | 0.21 |
| PC1 | 1.24 | 0.28 | 0.66 | 0.43 | 0.05 | 0.83 |
| PC2 | 1.01 | 0.33 | **4.77** | **0.04** | 2.13 | 0.16 |

**Note:** N * P: interactions between N addition and P addition; SOC: soil organic carbon; Avail P: available P. F: B indicates the ratio of fungal to bacterial PLFAs. G^+^: the proportion of gram-positive bacterial PLFAs; G^-^: the proportion of gram-negative bacterial PLFAs; Fungi: the proportion of fungal PLFAs; AM: the proportion of AM fungal PLFAs; Actino.: the proportion of actinomycetes PLFAs. Results are from two-way factorial ANOVA for the soil microorganism variables.
